# On the Necessity and Importance of the Accoucheur's Profession

**Published:** 1818-07

**Authors:** Thomas Wales


### *For the London Medical and Physical Journal.*

### *On the Necessity and Importance of the Accoucheur's Profession;* by THOMAS WALES, Esq.

HAVING been in the practice of Midwifery thirty years, I feel great interest in whatever relates to that important branch of the medical profession; of course, the correspondence that commenced by Dr. Kinglake's *Remarks on Obstetric Practice, and was successively carried on by Mr. Wayte, Mr. Atkinson, Dr. Adams, Dr. Merriman,† and others, not only arrested my attention at that period, (now more than two years ago,) but left an impression which can never be entirely removed.

The point in question was so ably discussed on the part of those who maintained the necessity of that branch of the profession remaining in the hands of male-accoucheurs, that I shall not enter into it farther than to observe, that although a great majority of labours may take place and be happily terminated without any necessary interference with the efforts of nature, yet I have never met with a female in any rank of life, who, having known the difference between the assistance or support rendered by male and female practitioners in perfectly natural cases, have not most unequivocally declared in favour of the former: and, were the choice left to the attending relations or neighbours, whose anxiety on these occasions is in proportion to the lack of efficient assistance, I am confident they would, without any exception, urge the propriety of having a man-midwife.

It must be very evident to the general reader, that Dr. Kinglake had never been in the habit of attending women in labour; still, some of his remarks are very judicious:—"Suitable delay and proper confidence in the resources of nature, are powerful auxiliaries in parturient difficulties." Again, "The practical maxim should be to wait sufficiently long clearly to ascertain that the accustomed competency of nature to the task is thwarted by either mechanical or physiological obstacles imperiously requiring the interposition of art." One of your correspondents in No. 203, has related the case of "a lady aged 40, who had been attended by a midwife, and was suffered to remain undelivered for at least twenty-four hours after the ear of the child might be felt; and, for that space of time, the head had not moved nearer the os externum: the pains had now nearly ceased; pulse

---

* Vide No. 200. † No. 202.

extremely weak; and countenance ghastly. The operation of embryotomy was performed in the presence of another practitioner, as the only means of giving the patient the smallest chance; in this way she was soon delivered of a very large child. It was, however, too late, she expired the following day."

Your correspondent does not say, whether he had ascertained that the child was dead previous to his operation; but I take for granted that was the case, as it is an axiom in the practice of midwifery, that whenever an ear can be felt, the forceps may be successfully applied. Indeed, in the same communication, he gives a reference to p. 161 of the London Practice of Midwifery, which I will copy:—" In a consultation that was held in a case of this kind, it was agreed, that nature certainly would be able to deliver the woman; she, therefore, was not interfered with. She did deliver herself, but lost her life by it; and that at a time when an ear was to be felt. This certainly was a case which required the use of the forceps which would have delivered her with safety."

I have a perfect recollection of the first case of midwifery I ever attended, which was in the Store-street Lying-in Hospital in the spring of 1788, being then a student and attending the lectures of Dr. Osborne and Mr. Clarke. I was called in my turn to attend a young woman: it proved to be a laborious case; and, after being confined to the ward all night, and the greatest part of the following day, I requested Mr. Clarke might be sent for, as I had felt an ear for many hours, yet the head came no forwarder. That gentleman soon arrived, and, finding the patient much exhausted, declared it to be one of those labours that required the use of forceps: they were applied, and the child was brought into the world in a very few minutes alive, and without the slightest injury. Since I have been in practice, I have been called to many such instances, where midwives of very considerable experience have found it necessary to send for a male-accoucheur. The forceps, in such cases, are generally applied without difficulty, and, in careful hands, always without injury to the mother or child.

I have been led to these remarks by an occurrence that has lately taken place in this town, which, however invidious it may appear, I am called upon to state as a public duty: in truth, it would have been transmitted two months ago, but that a legal opinion was requested to be had, and, pending the probability of a suit at law, it was not thought correct to publish a case which might eventually have gone before a jury. The husband, however, appears to have been frightened into submission to the event, (not being in circumstances to seek redress,) and the objection is, for the present, removed.

The case I am about to relate has, I think, no parallel,—even that alluded to in No. 108, p. 178, by Dr. Boys, man-midwife to the Westminster General Dispensary, which led to a capital indictment, differs materially: in the latter, the mother fell a sacrifice to a positive act of the accoucheur; whilst, in the former, every assistance that is used in all common cases of difficulty was unfeelingly and pertinaciously (I will not say ignorantly) withheld.

*Case.*—Sarah Bennet, aged 42, wife of Thomas Bennet, a carpenter, being, as she believed, at her full time, was taken with symptoms that denoted the approach of labour, on Saturday, February the 7th. The pains, though inconsiderable, were sufficient to convince her female neighbours that such was her situation. The usual appearance of a mucous discharge had also taken place, but, as the pains did not follow quickly, the accoucheur was not called till Tuesday the 10th.

On this day, the pains became very strong, and the membranes giving way, there was every prospect of her being delivered in a few hours; indeed, the liquor amnii escaping in small quantities with every pain, the women about her continually cheered her with that hope. Her medical attendant, however, without examining her, or, as is generally expressed, taking a pain, pronounced her to be not in labour, and left her immediately; but ordered some medicine, declaring it was an illness that required it.

On Wednesday the 11th, he called again, and then did examine; but, on this day, the pains were not so strong, the patient being much exhausted by the violence and continuance of pain the day before. He still pronounced it not to be labour, and left her after a few minutes, sending a fresh supply of medicine. After this day, Mrs. B. never felt the child, but continued night and day to have regular pains; and the remaining part of that week was passed in a state only to be described by the attendants.

The liquor amnii, and other discharges, had now (15th) obtained a very disagreeable fœtor, and she became an object loathsome to herself and those about her: had been visited every day by the accoucheur, and had a daily supply of medicine, but was never free from pain half an hour, so that she arose from her bed only to be refreshed by a change of position. The cloths were frequently shown to the practitioner, and every hope was expressed by the attendants that he would assist the suffering and apparently sinking patient.

This scene continued till Tuesday the 17th, when Nature again made great efforts to relieve herself of her burthen. On this day, other and most respectable persons, hearing of her situation, called to see her, and assured her of their firm belief that she might soon be delivered with the assistance of her medical attendant, and accordingly advised his being sent for. He came at the summons, but declined any examination, saying to the patient, "You will do very well; but, perhaps, it may be another day or two before your child be born:" and left her as usual. After this day, her appearance became ghastly and truly distressing; nothing stayed on her stomach, neither nourishment nor medicine. On Wednesday the 18th, the pains became weaker. On Thursday the 19th, hiccup and vomiting alternated with regular pains, but still weaker. On Friday the 20th, she appeared to those about her to be sinking fast: convulsions came on towards the evening, and the practitioner was requested to see her; this was between ten and eleven at night; she was now in strong convulsions, and did not recover her senses in the intervals: still *no examination* took place, nor was any step taken to relieve her; and, notwithstanding the earnest solicitations of the attendants, he refused to remain with the patient, and left her, ordering a blister to be applied to her back. A physician from Margate, upon a visit in the town, was present at this interview; and, it is asserted in a printed paper, that renders this publication necessary, that he sanctioned the practice—*Credat Judæus.* Her friends only witnessed a whispering that took place, but can speak to nothing farther.

On Saturday morning, (the 21st,) between seven and eight o'clock, being no longer able to bear the distressing scene, and believing the woman to be dying, one of the persons in attendance came to my house (which is not more than fifty or sixty yards distant) and requested I would go with her and render my assistance. I found the poor creature convulsed and totally insensible, and had reason to apprehend she would not survive even a few minutes. I instantly dispatched a messenger for the forceps, having ascertained the head to be not only low down in the pelvis, but that the tumour in perinæo was very prominent: they were applied without any difficulty, and a full sized dead child was brought into the world in about ten minutes. The convulsions immediately ceased, and the attendants imagined the danger to be all over; but, I regret to say, the woman never recovered her senses, although she survived nearly forty-eight hours.

This is the substance of a case unparalleled, one would hope, in the annals of midwifery: the particulars could have been more fully related from the evidence of the women who were present, all of whom agree in every circumstance that is material to a faithful relation of the transaction.

I shall make no other comments for the present on this melancholy business farther than to state my firm belief, that, for the last four days of the labour, the head was well into the pelvis, and the perinæum in a state of distension; for, from Tuesday the 17th, the patient could not sit upon her seat, but supported herself by resting the sacrum on the edge of a chair, with her hands placed behind her. I am also warranted in advancing this opinion from the state of the soft parts, which, with some remarks on the urinary bladder, will be the subject of another communication.

I understand from the accoucheur* himself, that he means to justify the whole of the proceeding in a reply to this statement; of course, it is expected the editor will have no hesitation in giving it a place in the next Number of the London Medical and Physical Journal.

*Downham, Norfolk;*
*May* 11, 1818.

---

*For the London Medical and Physical Journal.*

### Dr. Ferguson *on the Prevention of Typhus.*†

* A specimen of this young man's talents for asperity of language and scurrilous bravado, has made its appearance in a printed paper, (as a circular): of course, I have treated it with silent contempt, and shall, with confidence, look forward to a liberal and dispassionate discussion of the foregoing case by your professional readers.—[We have taken the liberty to omit some expressions contained in the manuscript note, conceiving that they should be confined to such a paper as Mr. Wales refers to.—Edit.]

